# Transparency too little, too late? Why and how Health Canada should make clinical data and regulatory decision-making open to scrutiny in the face of COVID-19

**DOI:** 10.1093/jlb/lsaa083

**Published:** 2020-11-19

**Authors:** Sterling Edmonds, Andrea MacGregor, Agnieszka Doll, Ipek Eren Vural, Janice Graham, Katherine Fierlbeck, Joel Lexchin, Peter Doshi, Matthew Herder

**Affiliations:** Schulich School of Law, Dalhousie University; Schulich School of Law, Dalhousie University; Schulich School of Law, Dalhousie University; Schulich School of Law, Dalhousie University; Department of Political Science, Dalhousie University; Department of Pediatrics (Infectious Diseases), Dalhousie University; Department of Political Science, Dalhousie University; School of Health Policy and Management, York University; Department of Pharmaceutical Health Services Research, University of Maryland School of Pharmacy; Schulich School of Law, Dalhousie University; Department of Pharmacology, Faculty of Medicine, Dalhousie University

**Keywords:** pharmaceutical regulation, transparency, COVID-19, vaccines and drugs, pandemic

Hard-won gains in the transparency of therapeutic product data in recent years^1^ have occurred alongside growing reliance by regulators upon expedited review processes.^2^ The concurrence of these two trends raises fundamental questions for the future of pharmaceutical regulation about whether the institutionalization of transparency will foster improved oversight of drugs, biologics, vaccines, and other interventions, or else, provide cover for a relaxing of regulatory standards of safety, effectiveness, and quality.^3^ The urgency of the COVID-19 pandemic, however, has brought this tension into immediate and sharp relief. During the course of the global health crisis, regulatory bodies have markedly expanded the number and use of expedited review processes for COVID-19 therapies, and at the same time, the proliferation of misinformation about any potential SARS-CoV-2 intervention^4^ reveals the limitations of recently implemented transparency measures.

Over the course of the pandemic, a range of candidate ‘therapeutic products’ (ie pharmaceuticals, biologics, vaccines, and medical devices)^5^ have rapidly entered clinical trials. In some cases, these products have already entered clinical use despite weak evidence of safety and effectiveness.^6^ Meanwhile, ‘preliminary findings,’ disclosed by companies, researchers, government officials, and the media have obscured the value of SARS-CoV-2 targeting products, fueling hype and precipitating misunderstanding about the merits of the product in question.^7^ With disclosure of the evidence behind these experimental products forestalled until formal market approval under existing transparency mechanisms, the limitations of a point-in-time approach to transparency have been revealed during the course of the pandemic.^8^

In this article, we explain why transparency must be radically expanded in several ways. We argue that meaningful transparency in the context of COVID-19 requires that the clinical data behind SARS-CoV-2 interventions and the regulatory decisions made based on that data must be open to scrutiny. We also argue that transparency should be expanded to occur upstream during therapeutic product development and continue in an expanded manner throughout its lifecycle, beyond the point of regulatory approval, as knowledge about the product’s safety and effectiveness continues to evolve. And, while the argument we develop applies in principle across jurisdictions, we zero in on Canada in particular where recently enacted transparency laws provide ample authority to implement our recommendations. Specifically, we detail how Canada’s existing transparency laws can be deployed to ensure that data, which only the sponsoring company may hold during the research process, are made available^9^ and facilitate independent scrutiny of information held by sponsors and the regulator alike in order to improve judgments about the safety and effectiveness of SARS-CoV-2 interventions. We begin by setting out the mechanisms by which COVID-19 therapeutic products are being authorized for clinical study and use, then develop arguments about why greater transparency is warranted before finally presenting how to do so precisely under current Canadian law. We close by considering how added transparency might better assure public trust in regulatory agencies, such as Health Canada.

## I. CANADA’S EXPEDITED REGULATORY PATHWAYS FOR COVID-19 CLINICAL TRIALS AND CLINICAL USE

Regulators worldwide have mobilized existing and new temporary mechanisms to expedite clinical trial approval and facilitate access to therapeutic products with uncertain benefits and harms in order to combat COVID-19.^10^ In Canada, this involves three mechanisms (Table 1): two of which pre-date the COVID-19 pandemic, while the third has been developed as the global health crisis has unfolded.

**Table 1:** Regulatory mechanisms to expedite clinical trials and authorize clinical use of therapeutic products during the COVID-19 pandemic in Canada.

	Established measures		Temporary measures		
	Special Access Program	Access to Drugs in Exceptional Circumstances Regulations	Interim Order Respecting the Importation and Sale of Medical Devices	Interim Order Respecting Clinical Trials for Medical Devices and Drugs	Interim Order Respecting the Importation, Sale and Advertising of Drugs
What are the mechanisms?	Provides access to unapproved drugs to practitioner on behalf of patients with serious or life-threatening conditions when conventional therapies have failed, are unsuitable, or are unavailable.	Regulations allow for the importation of foreign authorized drugs, not approved in Canada that would help address an urgent public health need	Provides the Minister of Health with authority to initiate immediate action to rapidly approve medical devices to assist with the response to COVID-19	Provides the Minister of Health with authority to rapidly approve clinical trials to determine the safety and efficacy of medical devices and drugs targeting COVID-19	Providers the Minister of Health with a number of new, expedited pathways to authorize the importation and sale of COVID-19 targeting drugs
Who requests access to the drugs/trial?	Practitioner (Physician)	Federal, Provincial or Territorial public health official	Minister of Health	Sponsors	Sponsors, the Chief Public Health Officer of Canada, Health Canada
What is population intended to benefit from the measure?	Individual patients	Population under the public health official’s authority	General population of Canada	Clinical trial participants	General population of Canada
What procedural requirements apply?	Application comes from practitioner on behalf of a patient, and it is reviewed by Health Canada	Streamlined notification by a public health official to Health Canada	Requires Governor-in-Council, ie Federal Cabinet approval	Two-step process:	The process varies depending on the expedited pathway. The Interim Order defines a number of requirements for sponsors to ‘pre-position’ a drug for market entry while also putting into place a rolling application process for drug submissions; a new foreign authorization pathway; a mechanism for Health Canada to add a new indication to a previous drug approval; and, a streamline set of requirements to apply for an Establishment License in order to lawfully manufacture a drug.
				(i) Authorization to import or sell drug/device to be tested in clinical trials by the Minister	
				(ii) Conduct of clinical trial approval by Health Canada	
When can imported drugs be used, either clinically or in a trial?	As deemed by practitioner	Immediately	Immediately	After authorizations are issued and an approval from a Research Ethics Board is obtained	After authorization by Health Canada
How long will access to drugs be granted or is the Interim Order in effect?	Practitioner-based process for renewal of additional quantities, sub-sequent authorization is needed to treat additional patients	One year. Renewable based on urgent public health need	One year. Not Renewable.	For the duration of clinical trial or until authorization is suspended or revoked.	Authorizations issued are valid until expiry of the Interim Order. Health Canada is considering various options to minimize disruptions for the ongoing authorization of drugs upon the expiry of the Interim Order with the intent to implement transition measures to ensure products maintain their legal status as needed.
What are the criteria for access? For commencing a clinical trial?	Onus on the requesting practitioner to show safety and efficacy information of drug in order to have their request approved	Designated drugs must be previously approved in the USA, EU, or Switzerland to be eligible	Designated drugs must have been previously approved for use in Canada (must have a Drug Identification Number)	Onus on the applicant that the use of device/drug will not unduly affect the health and safety of CT subjects, users, or other persons; CT is not contrary to best interests of CT subjects; objectives of CT are achievable	The criteria vary depending on the expedited pathway.

The pre-existing pathways by which unapproved interventions can enter clinical use in Canada are the Special Access Program (SAP)^11^ and the Access to Drugs in Exceptional Circumstances (ADEC) pathway.^12^ The SAP grants access for individual patients on a case-by-case basis upon physician request.^13^ Prior to its conditional approval by Health Canada in late July,^14^ the antiviral drug therapy remdesivir had been accessed by individual physicians under the SAP for the treatment of at least a dozen patients.^15^ In contrast to the case-by-case nature of the SAP, the ADEC (which has not yet been invoked during COVID-19) allows Health Canada to authorize the distribution and use of drugs at a population level, provided that they have been previously approved by a regulator in the USA, European Union, or Switzerland.^16^

The third mechanism known as Interim Orders, which authorize the federal Minister of Health to make temporary changes to the standard regulatory framework, has emerged as the option of choice for Health Canada, presumably because of the efficiency and flexibility that it provides.^17^ Several Interim Orders have been enacted during the pandemic to date, including one that streamlines clinical trial authorization for both drugs and medical devices,^18^ another that facilitates (or expedites) the clinical use of medical devices,^19^ and, in September 2020, an Interim Order that creates several new ways for drugs (defined to include pharmaceuticals, biologics, and vaccines) to enter the Canadian market.^20^ Pursuant to these Interim Orders, a number of clinical trials, testing hydroxychloroquine, remdesivir, and several vaccines, have been authorized in record time (Supplementary File),^21^ and one medical device has been authorized for clinical use (although it was subsequently recalled).^22^ No therapeutic products have been authorized under the most recent Interim Order to date, but a number of its features are worth noting.

To begin, the Interim Order specifies three new pathways to authorization by Health Canada. One is an expedited authorization procedure that allows for a ‘rolling application’ in which the sponsor submits information to the regulator based upon an agreed-upon schedule.^23^ This mirrors rolling application processes elsewhere and, by reducing the amount of information required initially upon submission, is intended to trigger faster decision-making about whether the benefits of the therapeutic product outweigh its risks.^24^ Another pathway allows for authorization where the product has been previously approved by one or more trusted foreign regulators, opening up the list of such regulators from the three eligible regulators under ADEC to at least seven foreign regulatory authorities.^25^ Finally, the Interim Order also describes how Health Canada can, without waiting for an application from the sponsor, seek to expand the indication of a previously approved drug to encompass treatment for COVID-19.^26^

Secondly, the drug-focused Interim Order aims to prioritize the review of applications by a sponsor for a new or modified ‘establishment license’ in order to lawfully produce a COVID-19 drug.^27^ Granting the regulator the discretion to alter the requirements typically applied to establishment license applications, the Interim Order’s stated that aim is to equip Health Canada with the ‘agility to facilitate rapid access to COVID-19 drugs while mitigating risks’.^28^

The third notable change introduced by the Interim Order is the creation of a ‘pre-positioning’ option to allow a drug to be imported into Canada by an establishment license holder and be prepared for distribution prior to market authorization.^29^ To be pre-positioned, the Government of Canada must have a procurement contract in place with the sponsor in respect of the Covid-19 drug in question,^30^ and the Chief Public Health Official of the Public Health Agency of Canada must provide notice to the federal Minister of Health.

While Health Canada has, with the enactment of several Interim Orders, demonstrated a responsiveness to the urgency of the pandemic, it is notable that these efforts to expedite access to experimental COVID-19 drugs, vaccines, and other interventions have not been accompanied by parallel increases in transparency.^31^ Health Canada has released little to no information underlying any of its therapeutic clinical trial authorizations,^32^ including relevant pre-clinical studies, clinical data from trials conducted abroad, and crucial details about the design of proposed clinical trials, such as clinical trial protocols, which specify inclusion/exclusion criteria for participants, primary and secondary outcomes that will be used to assess the product’s safety and efficacy, randomization and blinding procedures to ensure the integrity of the findings, and a variety of other information. A published summary of Health Canada’s decision to approve remdesivir, moreover, raises more questions than it answers given that the sponsor (Gilead Sciences) has yet to provide CSRs—the key document that regulators rely upon to assess safety and efficacy—for any of the COVID-19 remdesivir clinical trials.^33^ And, while several publicly available lists (identifying, for example, any applications that the regulator has received for marketing authorization against Covid-19^34^) were launched in conjunction with the September 2020 Interim Order, no meaningful changes were made to the regulator’s approach to transparency. On the contrary, Health Canada will only release data pertaining to a therapeutic product’s safety and effectiveness at the point of authorization.^35^ Given that the regulator has, in one case, reduced the level of information required for authorization (eg granting Gilead approval for remdesivir even in the absence of CSRs) and relaxed when information is due pursuant to the Interim Order (by creating a rolling application process for all sponsors), there is reason to worry that Health Canada may not possess much data to share. Before outlining how the regulator can take a more proactive and dynamic approach to transparency under Canadian law, we explain in depth why the absence of transparency during the research and development process and beyond approval may precipitate a range of harms in the context of COVID-19.

##  II. HARMS STEMMING FROM A LACK OF TRANSPARENCY SURROUNDING EXPERIMENTAL COVID-19 INTERVENTIONS

Poor data transparency, in any circumstance, poses risks to trial participants and patients, jeopardizes clinical trial quality,^36^ and has the potential to undermine trust in health professionals, pharmaceutical manufacturers, and regulatory decision-making.^37^ There is also some evidence that a perceived lack of transparency is one of the drivers of ‘vaccine hesitancy’.^38^ Many have therefore long agitated for greater transparency across the entire spectrum of pharmaceutical research and regulation, with some notable successes, including the creation and uptake of clinical trial registries such as ClinicalTrials.gov;^39^ the development of searchable databases documenting financial relationships between physicians and sponsors;^40^ and the passage of a variety of laws and policies meant to condition compliance with nascent transparency norms.^41^ Transparency remains a work in progress^42^ but the extent to which the treasure trove of previously undisclosed data from clinical trials, which the EMA and Health Canada now release upon product approval,^43^ will be regularly subject to independent scrutiny which is uncertain.^44^ It has the potential for transformative engagement by actors beyond the nexus of regulator and sponsor in the work of pharmaceutical governance, in theory, involving diverse expertise and relevant publics in the product’s appraisal.

Even so, the strides toward transparency that have been made in the pharmaceutical sphere in the recent years have diminishing value in the context of COVID-19, where experimental interventions are being rapidly trialed and/or entering clinical use through various expedited regulatory pathways. Delaying disclosure of the data underlying these regulatory decisions until the point of approval may be too late to guide rational decision-making given the misinformation available online about remedies that are not the subject of a clinical trial and, at the same time, intense political pressure to demonstrate progress towards a cure even if the intervention’s scientific merits are uncertain.^45^ In this particular context, transparency needs to happen upstream, prior to the therapeutic product authorization or approval, and across the product lifecycle, in order to mitigate or avoid several significant harms.

First, consider the potential harms to trial participants. Several trials authorized under the Interim Order pertaining to clinical trials aim to truncate or combine the different phases of a trial. This poses increased risks to trial participants when the safety of the intervention—normally first tested in a small group of healthy volunteers as part of a Phase 1 trial^46^—remains unknown as the larger Phase 2 trial, meant to assess efficacy in a larger sample of participants, begins. Results from a small Phase 1 trial in China of the COVID-19 vaccine candidate, known as Ad5-nCoV, sponsored by CanSino Biologics Inc.,^47^ likely served as the basis for authorizing a larger Phase 2 trial in China,^48^ as well as a combined Phase 1/2 trial in Canada.^49^ Given the lack of public access to data from the Phase 1 trial, or to the template consent forms used for the combined Phase 1/2 trial in Canada, it is not possible to assess whether patients will be informed of these risks, or what risk mitigation measures, if any, were built into the design of the larger trial.^50^ Rather than reviewing the trial protocol for consistency with ethical norms, Health Canada relies heavily on the prior approval of a Research Ethics Board (known as an Institutional Review Board in the USA) when deciding whether to authorize a trial.^51^ With a stated goal of authorizing trials within 14 days under the Interim Order, there is likely immense pressure upon local, under-resourced research ethics boards to sanction the trial’s design as quickly as possible. Embedded within the very institutions in which the research is being conducted, Canadian research ethics boards have routinely failed to apply and enforce ethical requirements in ordinary times,^52^ a reality potentially exacerbated by COVID-19. Public access to the trial consent forms could help train attention upon the risks endured by trial participants while also ensuring that consent forms are updated as risks associated with a particular COVID-19 candidate are newly identified.^53^ For instance, a participant in a trial of a vaccine under development by researchers at Oxford and AstraZeneca experienced severe neurological symptoms.^54^ But it is unclear whether that potential risk was incorporated into consent processes when trials of the vaccine resumed.

Further, the prospect of running ‘challenge trials’ in which a healthy volunteer is intentionally exposed to SARS-CoV-2, in an effort to more efficiently assess the effectiveness of the experimental candidate, underscores the importance of informed consent.^55^ There has been growing interest in challenge trials, marked by a grass roots campaign to enroll in such a trial were one to be initiated despite the absence of existing treatments for SARS-CoV-2, over the course of the pandemic.^56^ To ensure that the public is confident that participants are adequately informed of the risks involved, template consent forms used in the course of the trial should be publicly available, especially in light of lax regulatory oversight of clinical trials of late.^57^

Second, a lack of transparency stands to undermine the quality of clinical studies if the trials are not designed to answer the most pressing public health questions. For example, vaccine trials are expected to determine specific outcomes, eg the potential to reduce the incidence of COVID-19 infections and related complications, hospitalizations, or deaths.^58^ But without access to the trial protocol, which registries like ClinicalTrials.gov do not provide, it is impossible to assess whether the trial design can achieve robust results. For instance, one of the largest international COVID-19 vaccine trials, involving a novel mRNA vaccine, seeks to enroll 30,000 participants.^59^ Even if that target is met, the trial may not be large enough to assess whether the vaccine actually reduces the rates of hospitalization—one of the study’s secondary outcome measures—due to the infrequent rate of COVID-19 hospitalization in the general population.^60^ Several trials have already terminated early as new cases of COVID-19 waned in the region; new trials must have a viable plan for enrolling sufficient numbers.^61^ But without consistent transparency of study documents such as trial protocols or correspondence between sponsors and regulators, the opportunity is missed for timely evaluation when inadequacies can in theory still be corrected.^62^ Trial design modification may be unlikely even though regulators have the authority to compel changes to study design, but—at a minimum—the public can be more informed about the potential weaknesses of a given study.

Notably, some trial protocols have—at the sponsor’s discretion—been publicly disclosed during the course of the pandemic, including both Moderna and Pfizer’s mRNA vaccine trials, which include approximately 30,000 and 44,000 participants, respectively, as well as AstraZeneca’s US trial of its adenovirus-based vaccine.^63^ The details of these protocols raise some critically important questions, in particular, about whether the integration of ‘interim analyses’ in the design of the trials (the Pfizer, Moderna, and AstraZeneca trials include 4, 2, and 1, interim analyses, respectively) may lead to the trials being halted or modified prematurely (eg administering the experimental vaccine to the control group) if the efficacy of the vaccine in question is trending in a positive direction.^64^ Precisely how those decisions will be made is not delineated in the protocols that have been made publicly available; rather, those decision will fall to ‘data monitory committees’ (also known as ‘data safety monitoring boards’ or ‘DSMBs’), which typically operate under conditions of strict secrecy in order to both prevent false hope based on preliminary data,^65^ and to shield their members from outside influence.^66^ But the basic point remains that, unless trial protocols are—as a rule—open to scrutiny (as opposed to only when sponsors see fit to disclose them) as well as other key materials such as the ‘charters’ used by DSMBs to guide their decisions about stopping or modifying an ongoing trial (either because a safety issue has arisen or the intervention is showing significant therapeutic promise), it is not possible to fully interrogate the quality of COVID-19 trial designs and the reliability of trial outcomes. And, in the absence of trial protocol and DSMB charter transparency, before full data from the trial is released, there is a significant risk that downstream decisions by physicians about whether to administer the intervention in question to patients will be made without the benefit of strong evidence.

Third, the rush to promote partial, preliminary findings, has the potential to propagate misinformation. That is the main reason why data that are considered by DSMBs during the course of a trial are normally kept confidential.^67^ Over the course of the pandemic, however, sharing preliminary data has become the norm, not the exception. And while preprints have become an important vehicle for rapidly sharing scientific findings, they also have the potential to be misinterpreted or accepted as accurate before they have been subjected to rigorous peer review.^68^ Without the benefit of a peer-reviewed publication, let alone access to the underlying data which provide a more comprehensive record of a trial’s results,^69^ there is a significant chance that the risk–benefit profile of a given product will be misunderstood by both clinicians and the public.

Consider, for example, remdesivir’s path to market. No trial designs were publicly available before participant enrolment began. Weeks before ‘preliminary findings’ were published in high-profile journals,^70^ details of the drug’s purported benefits began to appear in the media, which Gilead promoted by press release.^71^ US government officials, specifically Dr Anthony Fauci, suggested remdesivir would become the ‘standard of care’ treatment for COVID-19.^72^ Trial details emerged, only after these press releases and announcements, raising significant concerns including the ‘serious methodologic error’ of prematurely censoring data of deceased patients, as well as design weaknesses such as lack of blinding and control, and limited sample size which may have produced unreliable results.^73^ Yet, the US government guaranteed that it would purchase virtually all of Gilead’s supply through September,^74^ fueling a global demand for the drug^75^ even though the evidence at the time suggested only moderate clinical benefit (eg reducing recovery time from 15 to 11 days)—a finding which the World Health Organization’s SOLIDARITY trial did not corroborate.^76^

Fourth, limited transparency—coupled with politicized and expedited regulatory decision-making—risks significant patient harm. Early adoption of remdesivir may, for instance, carry significant opportunity costs, halting or slowing the investigation of other treatments that could eventually prove more effective against SARS-CoV-2 because of patients’ reluctance to risk the chance of receiving a placebo in a new trial when there is an apparently effective drug already available. The United States’ Food and Drug Administration (USFDA) premature decision surrounding the anti-malarial drugs chloroquine and hydroxychloroquine is also illustrative. In March 2020, the USFDA granted ‘Emergency Use Authorizations’ (EUAs) for the two drugs to treat hospitalized COVID-19 patients.^77^ Numerous news sources, including Presidential tweets, referred to hydroxychloroquine’s emergency authorization as an ‘approval’ which sowed confusion and inflated perceptions of safety and efficacy.^78^ Subsequent randomized trials of hydroxychloroquine in hospitalized COVID-19 patients reported no evidence of benefit, and the EUA was subsequently revoked by the USFDA in mid-June, citing a lack of efficacy and concerns over side effects.^79^ Lack of transparency around the EUA prevented independent scrutiny of the USFDA’s initial determination that ‘the totality of scientific evidence’ made it ‘reasonable to believe that [the drugs] may be effective in treating COVID-19’.^80^

Under constant pressure from the Trump administration, the USFDA is holding firm to its stated standard of not authorizing a vaccine—even on an emergency basis—until at least two months have elapsed since trial participants have received the vaccine in question, and its observed efficacy meets or surpasses a pre-defined threshold.^81^ If and when an EUA is granted, it will be critical to ensure that the knowledge which continues to accumulate during the remainder of the trial is proactively and publicly shared post-authorization. The worry that underpins this call for continuous data transparency is two-fold: if a vaccine appears sufficiently effective, the DSMB may elect to allow it to be administered to participants in the control arm of the trial, nullifying the trial’s randomized design; or, even if no changes to the trial design are made, many participants will withdraw from the study once the decision has been made to grant an EUA.^82^ In either eventuality, the integrity of the trial’s results will be jeopardized. Therefore, ensuring that any changes made to trial designs and the trial results are open to scrutiny, both as the trial proceeds and after an EUA is granted, is essential. On a literal reading of the Interim Order issued in September, it appears that Health Canada can rubberstamp an EUA granted by the USFDA through the expanded foreign authorization mechanism that was introduced.^83^ Whether the Canadian regulator does so, or utilizes the expedited mechanism by which remdesivir was authorized in July, making trial designs and results more transparent may be key to countering public perceptions that COVID-19 interventions are as risky as they are rushed.^84^

##  III. HEALTH CANADA’S CURRENT APPROACH TO DATA DISCLOSURE AND HOW TO IMPROVE IT DURING COVID-19

Canada’s regulator has a long history of secrecy, electing to keep data confidential as a matter of practice.^85^ The introduction of ‘Vanessa’s Law’ in 2014,^86^ however, promised fundamental change. Adding a variety of patient safety measures to Canada’s *Food and Drugs Act*, including the power to unilaterally recall drugs from the market and compel acute care hospitals to share adverse event data with the federal regulator,^87^ one of the express purposes of Vanessa’s Law was to ‘promote greater confidence in the oversight of therapeutic products by increasing transparency’.^88^ To achieve that goal, several new legal authorities were added to the legislation, which, taken together, positioned Canada to become a global leader in transparency.^89^

Implementation of these transparency provisions has been gradual,^90^ but with the launch of Health Canada’s ‘Clinical Information Portal’ in March 2019 significant progress has been made.^91^ To date, the Canadian regulator has published important safety and efficacy data related to 75 drugs (including biologics and vaccines) and 15 medical devices. Some data are notably exempt from disclosure, including Case Report Forms (CRFs) that document outcomes for individual trial participants, and post-marketing experience in other jurisdictions.^92^ Still, a wide range of other data, including consent forms, outcome measures, blinding and randomization procedures, statistical analysis plans, and trial results are readily accessible via the Portal, which extends not only to interventions that have been approved (or rejected) since the Portal’s launch online, but also previously approved/rejected interventions.^93^ According to one analysis, Health Canada’s Portal offers the broadest scope of, and most timely access to, clinical data relative to the EMA and the USFDA.^94^

COVID-19, however, has revealed significant limitations to Health Canada’s newfound transparency. The first limitation concerns timing. Currently, disclosure of trial design and safety and efficacy data generated during human trials is sequestered by a specific point-in-time, ie if and when Health Canada formally approves or rejects a product.^95^ As a result, most data pertaining to an experimental intervention are treated as ‘confidential business information’ (CBI) (Table 2) before a regulatory decision is reached. To its credit, the recent Interim Order extends this point of disclosure to include authorizations granted under the Order.^96^ But in the context of COVID-19, trials are being rapidly authorized and misinformation about merits of various experimental interventions is prevalent. Delaying disclosure of clinical trial designs, correspondence between regulators and sponsors about those designs, and the basis for crucial decisions to be made by DSMBs about whether to halt or modify a trial at the point of interim analysis until after the decision to authorize or approve the intervention pre-empts the correction of potential flaws in trial designs and limits the opportunity to build public understanding of the knowledge and uncertainties behind a given COVID-19 intervention.^97^

**Table 2:** Clinical Information that is treated as CBI until after authorization.

Types of information	Description
Tabular listing of all clinical studies	List of all clinical studies for all drugs and for all Class III and IV medical devices.
CSRs	Any CSRs, including methodological details, specifications, and validation information.
Reports of biopharmaceutic studies	Studies that evaluate the rate and extent of release of active substance from the medicinal product (PK and BA data)
Reports of studies pertinent to pharmacokinetics (PK) using human biomaterials	*In vitro* studies to assess PK using biological systems
Reports of human PK studies	*In vivo* PK studies
Reports on human pharmacodynamic (PD) Studies	Receptor binding, receptor sensitivity, post-receptor effects, and chemical interactions
Reports of efficacy and safety studies	All available completed or on-going safety/efficacy-related studies on the drug in proposed and non-proposed indications.
Reports of post-marketing experience	Reports summarizing market experience (eg significant safety observations)
Literature references	Published articles, meeting minutes, regulatory advice.
Clinical overview	Clinical study overviews, including methodological details, specifications, and validation information.
Clinical summary	Summaries of all completed clinical studies and information submitted in support of the drug/medical device application for primary, secondary, or exploratory endpoints.

**Table 3:** Proposed information pertaining to COVID-19 interventions to be subject to targeted and public disclosure.

Legal Mechanism(s)	Type of Information	Disclosure Pathway or Platform	Timing of Disclosure
**Targeted Data Disclosure: DSMB Interim Analyses & IPD from Completed Trials**			
*Food & Drugs Act,* s 21.1(3)(c) Authorizing the disclosure of CBI without notice or consent from the person to whose business the information relates, for the purpose of protection or promotion of human health or the safety of the public.	Information about Product Ingredients, Materials, and Specifications	From Health Canada to Eligible Persons	Upon Request by Eligible Persons
*Food & Drugs Act,* s 21.1(3)(a) Authorizing the disclosure of CBI without notice or consent from the person to whose business the information relates, to a government body.	Interim Analyses Conducted by Data Safety and Monitoring Boards	From Health Canada to Eligible Persons	Upon Request by Eligible Persons
*Food & Drugs Act*, s 3.3 Creating a duty to publicize clinical trial information, subject to the regulations.	Anonymized Patient-Level Data from Completed Trials	From Sponsors to Health Canada and, in turn, Eligible Persons with an undertaking pursuant to the *Privacy Act* to preserve personal information	Upon Request by Eligible Persons
**Public Disclosure: Consent Forms, Trial Protocols, Clinical Study Reports & Regulatory Decisions**			
*Food & Drugs Act,* ss 30(1.2)(d.1) & 30(1.2)(d.2) Authorizing the creation of regulations specifying when CBI ceases to be confidential and allowing the disclosure of such information.	Consent Forms for Trial Participants (Previously Completed Trials Submitted as part of Clinical Trial Authorization Application)	From Health Canada via the Clinical Information Portal to the Public	Within 15 Days of Clinical Trial Authorization
*Food & Drugs Act*, s 3.3 Creating a duty to publicize clinical trial information, subject to the regulations.	Consent Forms for Trial Participants (New Trials)	From Health Canada via the Clinical Information Portal to the Public	Within 15 Days of Clinical Trial Authorization
	Complete Study Protocols (Previously Completed Trials Submitted in Support of new Clinical Trial Application)	From Health Canada via the Clinical Information Portal to the Public	Within 15 Days of Clinical Trial Authorization
	Pre-clinical Study Data	From Health Canada via the Clinical Information Portal to the Public	Within 15 Days of Clinical Trial Authorization
	Complete Study Protocols (New Trials)	From Health Canada via the Clinical Information Portal to the Public	Within 15 Days of Clinical Trial Authorization
	Correspondence Between Health Canada and Sponsor re: Clinical Trial Designs	From Health Canada via the DHPR to the Public	Within 15 Days of Clinical Trial Authorization
	Trial Outcomes (New Trials)	From Sponsors to Health Canada and in turn the Public via the Clinical Information Portal	Within 60 Days of Product Authorization
	Clinical Study Reports (New Trials)	From Sponsor to Health Canada and in turn the Public via the Clinical Information Portal	Within 60 Days of Product Authorization
	Health Canada Reviews and Rationale for Authorization	From Health Canada via the DHPR to the Public	Within 60 Days of Product Authorization
	Internal Communications and Memoranda between Health Canada and Sponsors	From Health Canada via the DHPR to the Public	Within 60 Days of Product Authorization

Secondly, even when disclosure is permitted, Health Canada may not possess the data to make it available. In ordinary circumstances, Health Canada does not, for example, require sponsors to submit CRFs unless a serious adverse event has occurred in the course of a study.^98^ Unless the regulator compels companies to submit and/or disclose such patient-level data, it is not possible for independent researchers to carry out a re-analysis of a trial—a labor-intensive act of independent scrutiny that continues to be undervalued but has, at times, revealed deep inconsistencies between published studies and what the trial actually found.^99^ In the context of the current pandemic, however, Health Canada has shown a willingness to accept data on a piecemeal basis and even approved one drug (remdesivir) without the benefit of CSRs. Until those CSRs are submitted to Health Canada, little to no information about remdesivir’s safety and efficacy is likely to be published via the Portal. With the Interim Order’s introduction of a new expedited ‘rolling application’ process, it is unclear how much data Health Canada will have to release at the time of market authorization.^100^

Finally, the Portal does not incorporate product safety and efficacy data that accumulates post-approval.^101^ Health Canada, like the USFDA, tends to provide little information about what post-market studies must be completed, sponsors’ progress in fulfilling them, or the regulator’s evolving understanding of the product’s safety and efficacy in light of those post-market studies.^102^ It is anticipated that many of the trials investigating COVID-19 drugs and vaccines will continue following authorization, especially if authorization occurs after an interim analysis; however, unless that post-authorization evidence is incorporated into the Portal, the evidence that will be open to independent scrutiny may represent only a fraction of what is known.

Fortunately, Canada’s *Food and Drugs Act* provides several options to address the foregoing gaps, in turn, improving the overall transparency of the data and decision-making process surrounding SARS-CoV-2 interventions. Depending on who possesses the information at a particular interval, different legal mechanisms can be invoked (or modified by way of an Interim Order) to ensure that data disclosure occurs through one of the several existing information-sharing platforms. Furthermore, transparency need not always mean disclosure to the public writ large; instead, the approach to disclosure should be specific to the type of information involved, purpose driven, and complemented by the necessary resources (Table 3).

###  III.A. Targeted Data Disclosure: DSMB Interim Analyses and Individual Patient Data from Completed Trials

Existing Canadian law grants the federal Minister of Health the discretion to disclose information that is deemed CBI to eligible persons, that is, persons who are engaged in the protection or promotion of human health and/or public safety,^103^ provided they intend to use the CBI for a health- or public safety-related purpose rather than a commercial one.^104^ This legal authority could be used, in the context of COVID-19, to share a number of different data with independent researchers before a decision is made to authorize a COVID-19 intervention for broader clinical use. Upon request from an eligible person (or government body),^105^ for example, the Minister could disclose completed COVID-19 pre-clinical studies and trials conducted outside Canada that are submitted to Health Canada as part of an application to conduct a new clinical trial.

With several candidate vaccines now under study in large, Phase 3 clinical trials, this same discretionary authority could also be invoked to share the Interim Analyses conducted by DSMBs and shared with the regulator. As described above, it is plausible that such Interim Analyses will be used to justify an expedited authorization of a vaccine. While the trials will likely continue after such an authorization, any decision by a DSMB to alter a trial should be open to scrutiny by independent trialists and other researchers.

Finally, at a later point in time, when the trials are completed, Health Canada can make the individual patient data (IPD) available to eligible researchers in order to validate the safety and efficacy findings that are reported by the sponsor. In the eventuality that Health Canada does not possess the necessary IPD (eg CRFs) from completed trials, an additional provision in the *Food and Drugs Act* can be invoked to compel sponsors to disclose that information—as prescribed by regulations—in respect of products that have been imported into Canada for the purpose of a clinical trial and/or received an authorization or approval from Health Canada.^106^ The regulator would need to define how that would occur using a new Interim Order and, in collaboration with other government agencies (eg Canadian Institutes of Health Research), marshal the resources to fund the labor-intensive work of re-analyzing and validating previous trial results using the IPD.^107^ Provided that the recipients of the IPD protect the privacy of trial participants’,^108^ allocating even 1–2 per cent of the >$850 million already earmarked for the development of SARS-CoV-2 interventions for independent assessment would appear to be a worthwhile investment.

###  III.B. Public Disclosure: Consent Forms, Trial Protocols, CSRs and Regulatory Decisions

Other types of data merit disclosure to the wider public. In order for the public to know that participants are being appropriately informed about the risks of participating in rapidly designed and authorized clinical trials, template consent forms that serve to enroll participants should be publicly shared before trials begin and in the event that the forms are updated in response to an adverse event. Challenge trials, if sanctioned, must be accompanied by disclosure not only of the consent forms but also the regulator’s underlying ethical justification for authorizing a challenge trial design, given the absence of an effective treatment against SARS-CoV-2 in order to assuage public concerns about this type of trial.^109^

Where the goal is to improve clinical trial reliability and promote scrutiny of trial results as well as regulatory decision-making, Health Canada should leverage its existing Clinical Information Portal and Drug and Health Product Register (DHPR)^110^ to make these and other data publicly available. The Portal can serve to publicly share the pre-clinical and clinical evidence behind all clinical trial authorizations and all expedited authorizations granted pursuant to ADEC or the Interim Order. The DHPR can provide a repository for consent forms used in trials while also serving as a space for the regulator to document its rationale for each decision, including authorizing challenge trials, key correspondences with sponsors about trial designs, trial results, studies required to be carried out post-approval, and any other considerations that factored into the decision to authorize a product.

Currently, both the Portal and DHPR encompass information pertaining only to approved drugs and devices. However, Health Canada has broad authority to alter what information is considered CBI, and when to deem it no longer to be CBI in order to release the information. Amendments to the regulations, or an Interim Order, can be used to expand the information to be publicly disclosed to points upstream in the research process as well as after authorization as the evidence base continues to evolve.^111^ The challenge concerning the clinical evidence to be posted on the Portal is timing. Unlike pre-clinical studies and other data that the regulator possesses as part of an application to conduct a clinical trial, the main documentation summarizing the safety and efficacy findings for completed trials (ie CSRs) can take weeks or months to prepare. The example of remdesivir suggests that Health Canada is prepared to accept and approve a drug submission without CSRs and more complete data in hand, and the recent introduction of a rolling application process appears to codify that same approach for the remainder of the pandemic. Therefore, to improve scrutiny of clinical trial designs and findings, Health Canada should disclose all the information it has regarding new trials, especially trial protocols, within 15 days of trial authorization (the same time frame in which the regulator is reviewing trial applications during the pandemic). The regulator should also invoke its authority—similar to patient-level data—to expedite the preparation and transfer of CSRs of all completed trials to Health Canada.^112^ The regulator can then disclose these data to the public via the Portal within a reasonable time frame following trial completion (eg 60 days) as opposed to the current 120 days.^113^

Finally, the DHPR, which presently contains only dates of meetings and limited summaries of Health Canada’s interpretation of the evidence after approval,^114^ similarly requires expansion to provide a complete record of each regulatory decision. No change in law is needed for this, as the reasons for Health Canada’s decisions have long been theirs to share.^115^ The DHPR should include all substantive correspondence between the regulator and sponsors, particularly with respect to trial designs, as well as a detailed explanation about why a given intervention is being trialed, made available through ADEC, authorized under the Interim Order’s provisions, or approved through another pathway. The DHPR should also include any information pertinent to conditions attached to an approval, especially those pertaining to post-market studies, with frequent and timely updating as studies are designed and completed.^116^

##  IV. REAL-TIME TRANSPARENCY, COMPETING CONSIDERATIONS AND THE QUESTION OF TRUST

By expanding transparency in the above ways, the Health Canada can enable deeper, science-based deliberations regarding what is known about a COVID-19 intervention, as the evidence evolves, rather than at a single point-in-time. With one intervention already approved in Canada (remdesivir) and one or more vaccines approaching authorization probably within the next 3–6 months, the federal government may need to overcome additional barriers to knowledge sharing in order to scale up production of the most promising products as no one manufacturer likely has the capacity to produce enough doses to meet the world’s needs.^117^ This may entail issuing a compulsory license in order to allow other manufacturers to produce a patented vaccine^118^ as well as encouraging firms to share manufacturing know-how, proprietary adjuvants, and assays, which may be protected as trade secrets.^119^ The prior task, however, is to ensure that the evolving knowledge about COVID-19 product candidates is meaningfully and continuously opens to scrutiny.

In principle, rendering clinical and decision-making data open to scrutiny carries competing considerations but none appear persuasive. The first concerns compliance with international law. Canada, like other signatories to international treaties governing intellectual property rights, is required to protect data against unfair commercial use. That commitment is an effort to balance the interests of first-mover and generic firms within the industry as they compete for market share.^120^ In the context of COVID-19, however, this balancing can and has already been addressed in other ways. Specifically, the Interim Order passed in September stipulates that any product (and, by extension, its underlying data) that is authorized under the Order cannot be cited as a reference product by a generic firm in an effort to obtain regulatory approval.^121^ As such, sponsors that reach the market first will not face generic competition unless they are unable to meet market demand for their product; in any event, international law allows data to be protected against unfair commercial use in a variety of ways and, insofar as the data are being made openly available to verify its reliability, as opposed to supporting a competing product, such use should not be characterized as commercially unfair. Enhanced disclosure of the data behind a COVID-19 intervention would not, as a result, appear to violate Canada’s international commitments.

Secondly, enhancing the level of transparency will require resources. Health Canada has, however, already devoted substantial resources to the implementation of Vanessa’s Law; moreover, the Clinical Information Portal and DHPR are already in place and, taken together, could incorporate all of the information to be made publicly available. Prior to the pandemic, Health Canada developed a set of review procedures to mitigate the risk of proprietary or personal information from being inadvertently disclosed via the Portal. In the usual course, this process is expected to consume 120 days.^122^ Given the urgency of COVID-19 and the potential harms that flow from a lack of openness, an Interim Order designed to expand transparency might temporarily waive this review process altogether, allowing officials to efficiently post template consent forms, protocols, correspondence, CSRs, and decision letters to sponsors that accompany trial or product authorization. In contrast, the targeted disclosure of DSMB interim analysis data and anonymized IPD will require some resources, in part, to review requests with a view to ensuring that the intended recipients have the requisite capacity to provide independent scrutiny of the data in question. Allocating funding to support independent scrutiny will also be important if such targeted disclosures are to be completed in time to help inform patient understanding of a drug or vaccine’s safety and efficacy and, assuming its risk–benefit profile proves favorable, improve uptake in the population as a whole. With the perception that COVID-19 vaccines are being rushed by sponsors and regulators alike, and public vaccine hesitancy seemingly on the rise,^123^ providing funds for independent researchers to interrogate the data behind one or more vaccines would seem a sound investment.

In the end, there is no necessary relationship between transparency and trust. Sharing less information might, in theory, limit fears of potential adverse events. On the other hand, the current situation echoes government-driven vaccine races from the past, such as the one developed for the forecasted 1976 influenza pandemic, the side effects of which helped to propel anti-vaccination movements to this very day. A COVID-19 intervention that is administered to whole swaths of the world’s population—without full transparency about its safety and efficacy—may engender lasting distrust not only against COVID-19 vaccines but also a range of other infectious disease interventions with more established safety and efficacy profiles.^124^ The best way to prevent that outcome is to ensure high-quality clinical trials, independent scrutiny of the resulting findings, and an unprecedented level of regulatory candor about experimental COVID-19 interventions in real time. Enhanced transparency should be a marker of the intervention’s trustworthiness—an expression of the regulatory system’s effort to convey what is known, to open that knowledge and judgment up to outsiders, and to invite critical reflection about whether a particular drug or vaccine will help us to re-emerge from COVID-19.

## AUTHOR CONTRIBUTIONS

S.E. and M.H. conceived of the work. S.E. wrote the original draft of the manuscript. All of the authors contributed to the design of the work, revised it critically for important intellectual content, gave final approval of the version to be published and agreed to be accountable for all aspects of the work.

Funding: This project was supported by an external grant from the Canadian Institutes of Health Research (CIHR PJT 156256). The funder had no role in the design and conduct of the study; collection, management, analysis, and interpretation of the data; preparation, review, or approval of the manuscript; and decision to submit the manuscript for publication.

## COMPETING INTERESTS

Lexchin received payments for writing a brief in an action for side effects of a drug for Michael F. Smith, Lawyer and a second brief on the role of promotion in generating prescriptions for Goodmans LLP. He is a member of the Foundation Board of Health Action International. He receives royalties from University of Toronto Press and James Lorimer & Co. Ltd. for books he has written. Doshi has received travel funds from the European Respiratory Society (2012) and Uppsala Monitoring Center (2018); grants from the Laura and John Arnold Foundation (2017–21), American Association of Colleges of Pharmacy (2015), Patient-Centered Outcomes Research Institute (2014–16), Cochrane Methods Innovations Fund (2016–18), and UK National Institute for Health Research (2011–14); and is an editor at The BMJ and unpaid member of the Reagan-Udall Foundation for the FDA. Fierlbeck is currently receiving research funding from CIHR, SSHRC, the Nova Scotia COVID-19 Health Research Coalition, and the EU’s Erasmus+ funding authority, as well as book royalties from the University of Toronto Press, McGill-Queen’s University Press, Routledge, and the University of Manchester Press. Herder reported being a member of the Patented Medicine Prices Review Board, Canada’s national drug price regulator, and receiving honoraria from the Board for his service. No other competing interests were declared.

## Supplementary Material

Transparency_too_late_Supplementary_File_FINAL_lsaa083

## Footnotes

1 Matthew Herder & Peter Doshi, *Precedent pushing practice: Canadian court orders release of unpublished clinical trial data*, BMJ Blogs (July 19, 2018), https://blogs.bmj.com/bmj/2018/07/19/precedent-pushing-practice-canadian-court-orders-release-of-unpublished-clinical-trial-data/.

2 Audrey D. Zhang et al., *Assessment of Clinical Trials Supporting US Food and Drug Administration Approval of Novel Therapeutic Agents, 1995–2017,* 3(4) Jama Netw. Open e203284 (2020), DOI: 10.1001/jamanetworkopen.2020.3284.

3 Vulnerable: The Law, Policy and Ethics of COVID-19 (Colleen M. Flood, Vanessa MacDonnell, Jane Philpott, Sophie Thériault, & Sridhar Venkatapuram, eds., 2020); Kamran Abbasi and Andrew Herxheimer, *The European Medicines Evaluation Agency: Open to Criticism: Transparency Must Be Coupled with Greater Rigour,* 317(7163) BMJ 898 (1998)*.*

4 John Zarocostas, *How to Fight an Infodemic*, 395 (10225) Lancet 676 (2020), DOI: 10.1016/S0140-6736(20)30461-X.

5
*Protecting Canadians from Unsafe Drugs Act (Vanessa’s Law),* S.C. 2014, c 24; *Food and Drugs Act,* R.S.C. 1985, F-27.

6 See discussion of remdesivir and hydroxychloroquine, *infra*, notes 70–80 and accompanying text.

7
*Id*.

8 Regulatory agencies publicly disclose a great deal of information. The vast majority of data pertaining to the safety and efficacy of a drug or vaccine is disclosed after the intervention has been approved. The US Food and Drug Administration (USFDA), for instance, is legally obliged to publicly disclose the ‘approval package’, which contains all of the scientific reviews completed by various disciplines within the agency. These approval packages often contain a number of details pertaining to the clinical trials carried out by the sponsor in the course of developing the drug, although the actual Clinical Study Reports (CSRs) prepared in respect of those trials are not disclosed by the USFDA. For further details about USFDA’s approval packages and their value to public health, see Matthew Herder, Christopher J. Morten & Peter Doshi, *Integrated Drug Reviews at the US Food and Drug Administration—Legal Concerns and Knowledge Lost*, 180 JAMA Intern Med 629–630 (2020). Like the USFDA, Health Canada and the European Medicines Agency (EMA) disclose the bulk of safety and efficacy data pertaining to therapeutic products after a regulatory decision has been rendered. In those two jurisdictions, however, CSRs are the principal information that is disclosed. The reviews conducted by each agency are not published apart from high-level summaries of the decisions taken. It is also notable that the Canadian and European regulators also disclose such data in the event when the intervention is denied market entry. The USFDA, in contrast, treats such rejections as proprietary unless and until a drug is subsequently approved. For an in-depth, comparative review of these three transparency mechanisms, see Alexander C. Egilman et al., *Transparency of regulatory data across the European Medicines Agency, Health Canada, and US Food and Drug Administration* (under review).

9 For example, Health Canada authorized a SARS-CoV-2 targeting drug (remdesivir) solely on the basis of ‘study protocols and preliminary and/or topline results’. The CSRs that normally accompany a New Drug Submission to the regulator were apparently not provided. We discuss this and other examples of data that only the sponsor may have at the time in question and Health Canada’s legal authority to compel the production of that information. See *infra* note 33 and accompanying text.

10 Health Canada, *Health Canada’s regulatory response to COVID-19: Access to health products*, aem (2020), https://www.canada.ca/en/health-canada/services/drugs-health-products/covid19-industry/regulatory-response-health-product-access.html (last visited Oct. 27, 2020); Center for Drug Evaluation and Research, *Coronavirus Treatment Acceleration Program (CTAP)*, FDA (2020), https://www.fda.gov/drugs/coronavirus-covid-19-drugs/coronavirus-treatment-acceleration-program-ctap (last visited Oct. 27, 2020); European Medicines Agency*, First COVID-19 Treatment Recommended tor EU Authorisation,* EMA (June 25, 2020)*,*  https://www.ema.europa.eu/en/news/first-covid-19-treatment-recommended-eu-authorisation; Emergo, *Medical Device and IVD Emergency Use Routes in Europe*, Emergo by UL, https://www.emergobyul.com/services/europe/medical-device-and-ivd-emergency-use-routes-europe (last modified June 10, 2020).

11 Canada *Food and Drug Regulations*, C.R.C., c 870, ss C.08.010, C.08.011. The SAP program is analogous to the USFDA’s expanded access program. See Office of the Commissioner, *Expanded Access*, FDA (2020), https://www.fda.gov/news-events/public-health-focus/expanded-access (last visited Oct. 17, 2020).

12 Canada *Food and Drug Regulations* C.R.C., c 870, s C.10.001 (2).

13 See *Food and Drug Regulations*, C.R.C., c 870, s C.08.010.

14 See Health Canada, *Remdesivir Authorized with Conditions for the Treatment of Patients in Canada with Severe COVID-19 Symptoms*, Government of Canada (July 28, 2020), https://healthycanadians.gc.ca/recall-alert-rappel-avis/hc-sc/2020/73621a-eng.php (accessed July 28, 2020).

15 Tom Blackwell, *Canadian experts do not see Remdesivir as a COVID-19 killer: ‘This is not a silver bullet,’* National Post, May 1, 2020, https://nationalpost.com/health/more-data-supply-needed-before-making-promising-covid-19-drug-remdesivir-a-routine-part-of-treatment-in-canada-say-experts (last visited Oct. 27, 2020).

16 With the exception of the EMA’s approval of remdesivir, none of those jurisdictions have formally approved of an intervention against COVID-19 to date.

17
*Food and Drugs Act*, R.S.C. 1985, c F-2, s 30.1(1).

18
*Interim Order Respecting Clinical Trials for Medical Devices and Drugs Relating to COVID-19* (May 23, 2020), https://www.canada.ca/en/health-canada/services/drugs-health-products/covid19-industry/interim-order-respecting-clinical-trials-medical-devices-drugs.html.

19
*Interim Order Respecting the Importation and Sale of Medical Devices for Use in Relation to COVID-19* (Mar. 18, 2020), https://www.canada.ca/en/health-canada/services/drugs-health-products/drug-products/announcements/interim-order-importation-sale-medical-devices-covid-19.html.

20
*Interim Order Respecting the Importation, Sale and Advertising of Drugs for Use in Relation to COVID-19* (Sept. 16, 2020), https://www.canada.ca/en/health-canada/services/drugs-health-products/covid19-industry/drugs-vaccines-treatments/interim-order-import-sale-advertising-drugs.html (hereinafter September Interim Order).

21 See Health Canada, *Drugs and Vaccines for COVID-19: List of Authorized Clinical Trials*, Government of Canada (June 24, 2020) (accessed July 1, 2020).

22 Health Canada, *Spartan Cube Covid-19 System (2020-05-05) (Recalls and Safety Alerts),* Government of Canada (May 6, 2020), https://healthycanadians.gc.ca/recall-alert-rappel-avis/hc-sc/2020/72971r-eng.php.

23 September Interim Order, s 3(2).

24 One vaccine has already started this process. *Canada Begins Review of Oxford Coronavirus Vaccine Candidate*, Global News (Oct. 2, 2020), https://globalnews.ca/news/7373941/canada-coronavirus-vaccine-review-oxford-astrazeneca/.

25 September Interim Order, s 4.

26 September Interim Order, s 15.

27 September Interim Order, s 20.

28 Health Canada, *Information and Application Requirements for Drugs Authorized under the Interim Order: Guidance Document* (Sept. 25, 2020)*,*  https://www.canada.ca/en/health-canada/services/drugs-health-products/covid19-industry/drugs-vaccines-treatments/interim-order-import-sale-advertising-drugs/guidance.html#a225. (hereinafter September Interim Order: Guidance Document) The Interim Order provides COVID-19 drug applicants an exemption from the Regulations with the exception of certain requirements in Part A and Divisions 1, 1A, 2, 3, and 4 of Part C. This means that, for example, Health Canada may alter the requirements under Division 5 (Drugs for Clinical Trials Involving Human Subjects), such as the good clinical practices requirements under s C.05.010.

29 September Interim Order, ss 27–28.

30
*Prime Minister Announces Funding to Advance the Development of Canadian COVID-19 Vaccine Technologies,* Government of Canada (Oct. 23, 2020), https://pm.gc.ca/en/news/news-releases/2020/10/23/prime-minister-announces-funding-advance-development-canadian-covid.

31 We describe what and when information is currently made transparent in Canada in detail below.

32 Health Canada provides a list of clinical trials involving SARS-CoV-2 interventions that have been authorized on its website. As of Oct. 2020, 68 trials had been authorized. Health Canada, *Drugs and vaccines for COVID-19: Authorized clinical trials*, aem (2020), https://www.canada.ca/en/health-canada/services/drugs-health-products/covid19-industry/drugs-vaccines-treatments/list-authorized-trials.html#wb-auto-4 (accessed Oct. 27, 2020). Little to no information about the design of these trials is provided on the website, however, where the trials have been registered on ClinicalTrials.gov and additional information is publicly available. As discussed below, however, ClinicalTrials.gov does not include trial protocols or consent forms.

33 See Health Canada, *Regulatory Decision Summary—Veklury—Health Canada*, Government of Canada (July 27, 2020), https://hpr-rps.hres.ca/reg-content/regulatory-decision-summary-detail.php?lang=en&linkID=RDS00669; United States Food and Drug Administration, *FDA’s Approval of Veklury (Remdesivir) for the Treatment of COVID-19—The Science of Safety and Effectiveness*, FDA (Oct. 22, 2020), https://www.fda.gov/drugs/drug-safety-and-availability/fdas-approval-veklury-remdesivir-treatment-covid-19-science-safety-and-effectiveness.

34 Health Canada, *Drug and Vaccine Authorizations for COVID-19: List of Applications Received,* Government of Canada (Oct. 13, 2020), https://www.canada.ca/en/health-canada/services/drugs-health-products/covid19-industry/drugs-vaccines-treatments/authorization/applications.html; Health Canada, *Drug and Vaccine Authorizations for COVID-19: List of Authorized Drugs and Expanded Indications,* Government of Canada (Sept. 17, 2020), https://www.canada.ca/en/health-canada/services/drugs-health-products/covid19-industry/drugs-vaccines-treatments/authorization/list-drugs.html; Health Canada, *List of Foreign Drugs in Relation to The COVID-19 Pandemic,* Government of Canada (Sept. 17, 2020), https://www.canada.ca/en/health-canada/services/drugs-health-products/covid19-industry/drugs-vaccines-treatments/authorization/foreign-drugs.html; Health Canada, *List of New Drugs for Expanded Indication in Relation to the COVID-19 Pandemic,* Government of Canada (Sept. 17, 2020), https://www.canada.ca/en/health-canada/services/drugs-health-products/covid19-industry/drugs-vaccines-treatments/authorization/expanded-indication.html.

35 See Health Canada, *Public Release of Clinical Information: Guidance Document*, Government of Canada (Jan. 4, 2019), https://www.canada.ca/en/health-canada/services/drug-health-product-review-approval/profile-public-release-clinical-information-guidance/document.html#appendix-a.

36 By ‘quality’ we mean the trial’s ability to answer the *most important* public health or scientific questions. We use this term in contrast to clinical trial ‘reliability’, ie the trial’s ability to *accurately* answer the questions it has selected however important to public health they may be.

37 See Martin Letendre, *The Montreal Tuberculosis Outbreak Revisited*, Veritas R.B.*,* Apr. 11, 2016, https://researchethicssimplified.com/the-montreal-tuberculosis-outbreak-revisited/ (accessed July 27, 2020).

38 See Emilie Karafillakis et al., *HPV vaccination in a context of public mistrust and uncertainty: a systematic literature review of determinants of HPV vaccine hesitancy in Europe*, 15 Human Vaccines & Immunotherapeutics 1615–1627 (2019).

39 NIH: U.S. National Library of Medicine—ClinicalTrials.gov, https://clinicaltrials.gov/ (accessed Oct. 27, 2020).

40 Centers for Medicare and Medicaid Services, *Open Payments,*  https://www.cms.gov/OpenPayments (last modified Sept. 21, 2020, 2:52 PM).

41 See generally Susan F. Wood & Kristen L. Perosino, *Increasing Transparency at the FDA: The Impact of the FDA Amendments Act of 2007*, 123 Public Health Rep 527–530 (2008); Institute of Medicine, *Sharing Clinical Trial Data: Maximizing Benefits, Minimizing Risk (2015)*, https://www.nap.edu/catalog/18998/sharing-clinical-trial-data-maximizing-benefits-minimizing-risk (last visited Oct. 27, 2020); Sergio Bonini et al., *Transparency and the European Medicines Agency—Sharing of Clinical Trial Data*, 371 New England Journal of Medicine 2452–2455 (2014).

42 Gaps in the scope of these databases have been identified and the enforcement of transparency requirements has been sparse at times. Jennifer E. Miller, David Korn & Joseph S. Ross, *Clinical trial registration, reporting, publication and FDAAA compliance: a cross-sectional analysis and ranking of new drugs approved by the FDA in 2012*, 5 BMJ Open e009758 (2015); Monique L. Anderson et al., *Compliance with Results Reporting at*  ClinicalTrials.gov, 372 New England Journal of Medicine 1031–1039 (2015); A. P. Prayle, M. N. Hurley & A. R. Smyth, *Compliance with mandatory reporting of clinical trial results on*  ClinicalTrials.gov*: cross sectional study*, 344 BMJ d7373–d7373 (2012); Lev Facher, *NIH warns drug and device companies to post missing trial data*, STAT (2020), https://www.statnews.com/2020/08/04/nih-warns-missing-clinical-trial-data/ (last visited Oct 27, 2020).

43 We describe Health Canada’s approach in depth below.

44 Some notable examples of such independent scrutiny exist, but it is not yet clear whether such research will become the norm rather than the exception. For a powerful example of independent scrutiny, see Joanna Le Noury et al., *Restoring Study 329: efficacy and harms of paroxetine and imipramine in treatment of major depression in adolescence*, 351 BMJ h4320 (2015). Despite the value of such research to public health, some influential commentators continue to devalue reanalyses as derivative, secondary research. See eg Dan L. Longo & Jeffrey M. Drazen, *Data Sharing*, 374 N. Engl. J. Med 276–277 (2016).

45 Notably, a recent analysis found that President Trump is the single biggest source of misinformation during the pandemic. Sheryl Gay Stolberg & Noah Weiland, *Study Finds ‘Single Largest Driver’ of Coronavirus Misinformation: Trump*, The New York Times, Oct. 1, 2020, https://www.nytimes.com/2020/09/30/us/politics/trump-coronavirus-misinformation.html (last visited Oct 1, 2020).

46 Research indicates that participants in Phase 1 trials are often subject to mild or moderate harms. Rebecca A. Johnson et al., *Risks of phase I research with healthy participants: A systematic review*, 13 Clin. Trials 149–160 (2016).

47 See Feng-Cai Zhu et al., *Safety, Tolerability, and Immunogenicity of a Recombinant Adenovirus Type-5 Vectored COVID-19 Vaccine: A Dose-Escalation, Open-Label, Non-Randomised, First-In-Human Trial*, 395(10240) Lancet 1845 (2020), DOI: 10.1016/S0140-6736(20)31208-3.

48 See Feng-Cai Zhu et al., *Immunogenicity and Safety of a Recombinant Adenovirus Type-5-Vectored COVID-19 Vaccine in Healthy Adults Aged 18 Years or Older: A Randomised, Double-Blind, Placebo-Controlled, Phase 2 Trial,* Lancet Online (2020), DOI: 10.1016/S0140-6736(20)31605-6.

49 See ClinicalTrials.gov*, Identifier NCT04398147, Phase I/II Clinical Trial of Recombinant Novel Coronavirus Vaccine (Adenovirus Type 5 Vector) in Canada*, 2020, https://clinicaltrials.gov/ct2/show/NCT04398147 (accessed Jul. 27, 2020).

50 The Canadian trial was subsequently cancelled. Alex Cooke, *Canadian COVID-19 clinical trial scrapped after China would not ship potential vaccine | CBC News*, CBC, Aug. 26, 2020, https://www.cbc.ca/news/canada/nova-scotia/canada-china-covid-19-vaccine-trial-plug-pulled-1.5701101 (last visited Oct. 20, 2020).

51 Cite regs and also relevant provisions of the May IO. Anything different re REB approval? *Food and Drugs Regulations,* ss. C.05.006 (1)(c), C.05.008 (1)(c), C.05.010(d).

52 Jocelyn Downie, *The Canadian Agency for the Oversight of Research Involving Humans: A Reform Proposal*, 13 Accountability in Research 75–100 (2006).

53 Interestingly, one recent publication suggests that trial participants may be inadequately informed about the risk that the experimental vaccine may, paradoxically, “worsen COVID-19 disease via antibody-dependent enhancement.” The authors substantiate this claim through an examination of the clinical trial protocols that have been made publicly available, not the actual consent forms used in the trials. See Timothy Cardozo & Ronald Veazey, *Informed consent disclosure to vaccine trial subjects of risk of COVID-19 vaccines worsening clinical disease*, n/a International Journal of Clinical Practice e13795. Disclosure of the consent forms would enable independent assessment of whether participants were adequately informed of this risk.

54 Adam Feuerstein, *AstraZeneca CEO says participant had neurological symptoms, could be discharged today*, STAT, Sept. 9, 2020, https://www.statnews.com/2020/09/09/astrazeneca-covid19-vaccine-trial-hold-patient-report/ (last visited Oct. 25, 2020).

55 Lisa Tambornino & Dirk Lanzerath, *COVID-19 human challenge trials—what research ethics committees need to consider*, 16 Research Ethics 1–11 (2020).

56 Francoise Baylis & Landon Getz, *Rush to risky challenge trials is unethical*, Healthy Debate (2020), https://healthydebate.ca/opinions/risky-challenge-trials-unethical (last visited Oct 11, 2020).

57 Charles Piller, *FDA’s Own Documents Reveal Agency’s Lax, Slow, and Secretive Oversight of Clinical Research,* Oct. 1, 2020, Science, https://www.sciencemag.org/news/2020/10/fda-s-own-documents-reveal-agency-s-lax-slow-and-secretive-oversight-clinical-research.

58 Peter Doshi, *Will covid-19 vaccines save lives? Current trials aren’t designed to tell us*, 371 BMJ (2020), https://www.bmj.com/content/371/bmj.m4037 (last visited Oct. 25, 2020).

59 See Moderna, *Moderna Announces Expansion of BARDA Agreement to Support Larger Phase 3 Program for Vaccine (mRNA-1273) Against COVID-19*, July 26, 2020, https://investors.modernatx.com/news-releases/news-release-details/moderna-announces-expansion-barda-agreement-support-larger-phase (accessed July 27, 2020).

60 See U.S. Center for Disease Control*, Coronavirus Disease 2019 (COVID-19),* Aug. 7, 2020, https://www.cdc.gov/coronavirus/2019-ncov/covid-data/covidview/index.html (accessed Aug. 10, 2020).

61 See John David Norrie, *Remdesivir for COVID-19: Challenges of Underpowered Studies*, 395(10236) Lancet 1525 (2020), https://www.thelancet.com/lancet/article/s0140673620310230.

62 Peter Doshi, *Covid-19 vaccine trial protocols released*, 371 BMJ (2020), https://www.bmj.com/content/371/bmj.m4058 (last visited Oct. 25, 2020).

63 Denise Grady & Katie Thomas, *Moderna and Pfizer Reveal Secret Blueprints for Coronavirus Vaccine Trials*, The New York Times, Sept. 17, 2020, https://www.nytimes.com/2020/09/17/health/covid-moderna-vaccine.html (last visited Sep 21, 2020); Denise Grady, Katherine J. Wu & Sharon LaFraniere, *AstraZeneca, Under Fire for Vaccine Safety, Releases Trial Blueprints*, The New York Times, Sept. 19, 2020, https://www.nytimes.com/2020/09/19/health/astrazeneca-vaccine-safety-blueprints.html (last visited Sept. 21, 2020). Note that Astrazeneca UK trial protocol has not been released….

64 The acceptable threshold for efficacy depends upon when the data is being analyzed. For instance, Pfizer’s first interim analysis (when 32 participants in the trial have become infected) specifies an efficacy point estimate of 74%; at trial completion, ie when 150 or more participants test positive for COVID-19, the protocol specifies a threshold of 50%.

65 David L. DeMets & Susan S. Ellenberg, *Data Monitoring Committees—Expect the Unexpected*, 375 N. Engl. J. Med 1365–1371 (2016).

66 Rachana Pradhan, *These Secret Safety Panels Will Pick the COVID Vaccine Winners*, Kaiser Health News (2020), https://khn.org/news/these-secret-safety-panels-will-pick-the-covid-vaccine-winners/ (last visited Oct. 6, 2020).

67 DeMets & Ellenberg, *supra* note 65.

68 Adam Palayew et al., *Pandemic publishing poses a new COVID-19 challenge*, 4 Nat. Hum. Behav. 666–669 (2020).

69 Several studies have shown that published versions of trials present a very different picture than the evidence (from the same trials) that are submitted to regulators. See eg Erick H. Turner et al., *Selective publication of antidepressant trials and its influence on apparent efficacy*, 358 N. Engl. J. Med 252–260 (2008).

70 See Yeming Wang et al., *Remdesivir in Adults With Severe COVID-19: A Randomised, Double-blind, Placebo-controlled, Multicentre Trial*, 395(10236) Lancet 1569 (2020), DOI: 10.1016/S0140-6736(20)31022-9.

71 See Maryam Shah, *Remdesivir, Hailed as Potential COVID-19 Treatment, Gets Emergency U.S. FDA Green Light*, Global News, May 2, 2020, https://globalnews.ca/news/6895040/remdesivir-fda-emergency-use-covid-19/ (accessed July 11, 2020).

72 Sue Hughes, *Remdesivir Now ‘Standard of Care’ for COVID-19, Fauci Says*, Medscape*,* Apr. 29, 2020, https://www.medscape.com/viewarticle/929685 (accessed Aug. 5, 2020).

73 See Stefanos Bonovas & Daniele Piovani, *Letter to the Editor*, 382 N. Engl. J. Med. 2327 (2020); Gerd Fätkenheuer & Jen Lundgren, *Letter to the Editor*, 382 N. Engl. J. Med. 2327 (2020); Christian Hoffmann, *Letter to the Editor*, 382 N. Engl. J. Med. 2327 (2020); Jiayuan Wu, Bin Wu & Tianwen Lai, *Letter to the Editor*, 382 N. Engl. J. Med. 2327 (2020), DOI: 10.1056/NEJMc2015312.

74 See U.S. Department of Health and Human Services, *Trump Administration Secures New Supplies of Remdesivir for the United States [press release],* June 29, 2020, https://www.hhs.gov/about/news/2020/06/29/trump-administration-secures-new-supplies-remdesivir-united-states.html (accessed Aug. 5, 2020).

75 Sarah Boseley, *Global Shortage of Key Covid Drug Leads to NHS Rationing*, The Guardian, Oct. 6, 2020, https://www.theguardian.com/world/2020/oct/06/global-shortage-of-key-covid-drug-leads-to-nhs-rationing-remdesivir; Jing Luo, Gregg Gonsalves & Amy Kapczynski, *Treatments do not work if we cannot afford them: the global need for open and equitable access to remdesivir*, The BMJ (2020), https://blogs.bmj.com/bmj/2020/06/03/treatments-dont-work-if-we-cant-afford-them-the-global-need-for-open-and-equitable-access-to-remdesivir/ (last visited Oct. 19, 2020).

76 Interim results published on Oct. 15 found that remdesivir had “had little or no effect on overall mortality, initiation of ventilation and duration of hospital stay in hospitalized patients.” See “*Solidarity” Clinical Trial for COVID-19 Treatments*, (2020), https://www.who.int/emergencies/diseases/novel-coronavirus-2019/global-research-on-novel-coronavirus-2019-ncov/solidarity-clinical-trial-for-covid-19-treatments (last visited Oct. 17, 2020). Despite this, Canada has also made a procurement agreement for Remdesivir. Public Services and Procurement Canada, *Government of Canada Signs New Agreements to Secure Additional Vaccine Candidate and Treatment for COVID-19,* Sept. 22, 2020, https://www.canada.ca/en/public-services-procurement/news/2020/09/government-of-canada-signs-new-agreements-to-secure-additional-vaccine-candidate-and-treatment-for-covid-19.html.

77 See Denise Hinton, Chief Scientist, Food and Drug Administration, to Dr Rick Bright, Ph.D. Director Biomedical Advanced Research and Development Authority (BARDA), *Re: Request for Emergency Use Authorization for Use of Chloroquine Phosphate or Hydroxychloroquine Sulfate Supplied from the Strategic National Stockpile for Treatment of 2019 Coronavirus Disease* (Mar. 28, 2020), https://www.fda.gov/media/136534/download (accessed July 1, 2020).

78 Kashmira Gander, *FDA Says Hydroxychloroquine and Chloroquine Can Be Used to Treat Coronavirus*, Newsweek*,* Mar. 30, 2020, https://www.newsweek.com/fda-says-hydroxychloroquine-chloroquine-can-used-treat-coronavirus-1494925 (accessed July 9, 2020); Ronald Bailey R, *FDA Approves Emergency Use of Hydroxychloroquine and Chloroquine to Treat COVID-19,* Reason, Mar. 30, 2020, https://reason.com/2020/03/30/fda-approves-emergency-use-of-hydroxychloroquine-and-chloroquine-to-treat-covid-19/ (accessed July 9, 2020); Daniel Dale, *Fact Check: Trump Wrongly Claims FDA ‘Approved’ Drug Chloroquine to Treat the Coronavirus,* CNN*,* Mar. 19*,* 2020, https://www.cnn.com/2020/03/19/politics/fact-check-chloroquine-trump-fda/index.html (accessed July 9, 2020).

79 See U.S. Food and Drug Administration, *Coronavirus (COVID-19) Update: FDA Revokes Emergency Use Authorizations for Chloroquine and Hydroxychloroquine*, June 15, 2020, https://www.fda.gov/news-events/press-announcements/coronavirus-covid-19-update-fda-revokes-emergency-use-authorization-chloroquine-and (accessed June 29, 2020); U.S. Department of Health and Human Services, FDA, *Pharmacovigilance Memorandum*, May 19, 2020, p. 6, https://www.accessdata.fda.gov/drugsatfda_docs/nda/2020/OSE%20Review_Hydroxychloroquine-Cholorquine%20-%2019May2020_Redacted.pdf (accessed Jun. 29, 2020).

80 Denise Hinton, Chief Scientist, Food and Drug Administration, to Dr Rick Bright, Ph.D. Director Biomedical Advanced Research and Development Authority (BARDA), *Re: Request for Emergency Use Authorization for Use of Chloroquine Phosphate or Hydroxychloroquine Sulfate Supplied from the Strategic National Stockpile for Treatment of 2019 Coronavirus Disease*, Mar. 28, 2020, https://www.fda.gov/media/136534/download (accessed July 1, 2020).

81 Need to detail how threshold shifts depending on interim analysis, citing Pfizer protocol as an example. Jacqueline Howard & Maggie Fox, *FDA Wants two Months of Safety Data before Considering Covid-19 Vaccine*, CNN (2020), https://www.cnn.com/2020/10/06/health/fda-covid-vaccine-safety-data-bn/index.html (last visited Oct. 11, 2020); Vaccines and Related Biological Products Advisory Committee Oct. 22, 2020 Meeting Announcement—10/22/2020–10/22/2020, FDA (2020), https://www.fda.gov/advisory-committees/advisory-committee-calendar/vaccines-and-related-biological-products-advisory-committee-october-22-2020-meeting-announcement (last visited Oct. 11, 2020).

82 One of the companies with a vaccine in the late stages of development, Pfizer, has publicly stated that it will seek permission from the USFDA to allow participants in the control group to receive the vaccine in the event that an EUA is granted. See Donna Young, *US FDA expert panel raises concerns COVID-19 vaccines target mild disease*, S&P Global Market Intelligence (2020), https://www.spglobal.com/marketintelligence/en/news-insights/latest-news-headlines/us-fda-expert-panel-raises-concerns-covid-19-vaccines-target-mild-disease-60870744 (last visited Oct. 25, 2020).

83 September Interim Order, s 4(1).

84 Angela Jung, *New survey finds more Canadians are hesitant about getting a vaccine against COVID-19*, British Columbia (2020), https://bc.ctvnews.ca/new-survey-finds-more-canadians-are-hesitant-about-getting-a-vaccine-against-covid-19-1.5131271 (last visited Oct. 19, 2020); Ethan Hauser, Jill Cowan & Frances Robles, *Covid-19 Live Updates: American Public Grows Increasingly Wary of Virus Vaccines*, The New York Times, Oct. 20, 2020, https://www.nytimes.com/live/2020/10/20/world/covid-19-coronavirus-updates (last visited Oct. 20, 2020).

85 Matthew Herder, *Unlocking Health Canada’s cache of trade secrets: mandatory disclosure of clinical trial results*, 184 CMAJ 194–199 (2012) and, Matthew Herder, *Denaturalizing transparency in drug regulation*, 8 McGill J. L. & Health S57–S143 (2015).

86
*Protecting Canadians from Unsafe Drugs Act (Vanessa’s Law*), SC 2014, c 24.

87
*Vanessa’s Law*, ss 3, 5.

88
*Vanessa’s Law*, Summary. It is worth noting that the legislation did not originally include any measures to improve transparency. During the legislative process, though, the bill was amended to address the issue of transparency. Matthew Herder, *The Opacity of Bill C-17’s Transparency Amendments*, Impact Ethics (2014), http://impactethics.ca/2014/06/23/the-opacity-of-bill-c-17s-transparency-amendments/ (last visited Sept. 3, 2014).

89 See Egilman et al., *supra* note 9.

90 Herder & Doshi, *supra* note 1.

91 Health Canada, *Search for Clinical Information on Drugs and Medical Devices,* May 29, 2019, https://clinical-information.canada.ca/search/ci-rc.

92 Health Canada, *Guidance Document on Public Release of Clinical Information: Profile Page,* (Appendix A) Mar. 29, 2019, https://www.canada.ca/en/health-canada/services/drug-health-product-review-approval/profile-public-release-clinical-information-guidance.html.

93 It is worth noting that the Portal has yet to publish data in respect of a rejected drug submission.

94 Egilman et al., *supra* note 9. Importantly, though, the transparency of Health Canada’s decision-making relative to the USFDA and EMA is limited. The USFDA has, since 1997, published all of the scientific reviews connected to a given drug approval. The US agency has recently shifted its review process, threatening to undermine the transparency of its decisions. See Matthew Herder, Christopher J. Morten & Peter Doshi, *Integrated Drug Reviews at the US Food and Drug Administration—Legal Concerns and Knowledge Lost*, 180 JAMA Intern Med 629–630 (2020). For its part, the EMA publishes summaries of its decisions to approve and reject a drug from market entry. Meanwhile, Health Canada’s decision summaries tend to add little information to what is already in the public domain. For a comparison of the transparency of these three regulators’ decisions, see Matthew Herder, *Toward a Jurisprudence of Drug Regulation*, 42 The Journal of Law, Medicine & Ethics 244–262 (2014).

95 See *Food and Drug Regulations*, C.R.C., c 870, s C.08.009.2

96 The Guidance published in conjunction with the Interim Order states: “Health Canada will make publicly available the safety and efficacy evidence relied upon to issue an authorization under the Interim Order respecting authorization of drugs in relation to COVID-19.” Health Canada, *Information and Application Requirements for Drugs Authorized under the Interim Order: Guidance Document,* Sept. 17, 2020, https://www.canada.ca/en/health-canada/services/drugs-health-products/covid19-industry/drugs-vaccines-treatments/interim-order-import-sale-advertising-drugs/guidance.html#a12.

97 Health Canada’s Guidance with respect to the Portal explicitly notes that disclosure of interim analyses might bias trial results, therefore, decisions about whether to disclose interim analyses will be made on a case-by-case basis. Given how much misinformation is available about various COVID-19 interventions, however, the exclusion of interim analysis data from public disclosure marks a missed opportunity to inform wider audiences about the nuanced evidence under consideration. See Health Canada, *Public Release of Clinical Information: Guidance Document*, *supra* note 36.

98 In part for this reason, the Portal specifically excludes them from its scope of disclosure. See Health Canada, *Public Release of Clinical Information: Guidance Document*, *supra* note 36.

99 See eg Joanna Le Noury et al., *supra* note 45.

100 One sponsor has begun to avail of Health Canada’s rolling application process to date. See Health Canada, *Health Canada Begins First Authorization Review of a COVID-19 Vaccine Submission,* Oct. 2, 2020, https://www.canada.ca/en/health-canada/news/2020/10/health-canada-begins-first-authorization-review-of-a-covid-19-vaccine-submission.html. As of early Oct. 2020, the EMA has two rolling applications under review. Elena Kostadinova Dimitrova, *EMA starts second rolling review of a COVID-19 vaccine*, European Medicines Agency (2020), https://www.ema.europa.eu/en/news/ema-starts-second-rolling-review-covid-19-vaccine (last visited Oct. 20, 2020).

101 Apart from advisories intended to alert practitioners when a serious safety issue is identified, Health Canada publishes limited information about events in the post-market phase of a product’s lifecycle. The Drug and Health Product Register (described *infra* at note 110 and accompanying text.) includes a Post-Authorization Activity Table (PAAT) that provides, for example, very basic information about when new indications are granted. See eg Health Canada, *Summary Basis of Decision—Adynovate—Health Canada*, https://hpr-rps.hres.ca/reg-content/summary-basis-decision-detailTwo.php?linkID=SBD00340 (last modified Sept. 17, 2020). PAAT does not include information about ongoing clinical trials or other more substantive information about a product’s safety and efficacy.

102 See Joel Lexchin, *Health Canada’s Use of its Notice of Compliance with Conditions Drug Approval Policy: A Retrospective Cohort Analysis*, 49(2) Int. J. Health. Serv. 294 (2019); Joshua D. Wallach et al., Postmarket Studies Required by the U.S. Food and Drug Administration for New Drugs and Biologics Approved Between 2009 and 2012: Cross Sectional Analysis, 361 B.M.J. k2031 (2018), DOI: 10.1136/bmj.k2031.

103 See *Food and Drugs Act,* R.S.C. 1985, F-27, s 21.1(3)(c).

104 Health Canada, *Disclosure of Confidential Business Information*, May 7, 2019, https://www.canada.ca/en/health-canada/services/drug-health-product-review-approval/request-disclosure-confidential-business-information/disclosure-confidential-business-information.html.

105 See *Food and Drugs Act,* R.S.C. 1985, F-27, s 21.1(3)(a).

106 See *Food and Drugs Act,* R.S.C. 1985, F-27, s 3.3.

107 Health Canada has in the past tasked other government agencies with conducting research. The Drug Safety and Effectiveness Network has performed such research on multiple occasions. For an overview of this research, see Health Canada, *Horizontal Evaluation of the Drug Safety and Effectiveness Network (DSEN) 2014–15 to 2018–19*, aem (2020), https://www.canada.ca/en/health-canada/corporate/transparency/corporate-management-reporting/evaluation/drug-safety-effectiveness-network.html (last visited Oct. 27, 2020). Further Health Canada has already utilized this mechanism during COVID-19 See Canadian Institutes of Health Research, *DSEN Abstract*, Aug. 27, 2020, https://cihr-irsc.gc.ca/e/52124.html.

108 See Health Canada, *Guidance Document—Disclosure of Confidential Business Information under Paragraph 21.1(3)(c) of the Food and Drugs Act,* 2019, https://www.canada.ca/en/health-canada/services/drug-health-product-review-approval/request-disclosure-confidential-business-information/disclosure-confidential-business-information/guidance.html#a1.1 (accessed Jun. 30, 2020).

109 Here, it is important to note that although one treatment (remdesivir) has been approved by Health Canada to date, the evolving evidence casts significant doubt on its efficacy. Interim results published on Oct. 15 found that remdesivir (as well as three other interventions) had ‘had little or no effect on overall mortality, initiation of ventilation, and duration of hospital stay in hospitalized patients’. See ‘Solidarity’ clinical trial for COVID-19 treatments, (2020), https://www.who.int/emergencies/diseases/novel-coronavirus-2019/global-research-on-novel-coronavirus-2019-ncov/solidarity-clinical-trial-for-covid-19-treatments (last visited Oct. 17, 2020); and, WHO Solidarity Trial Consortium et al., *Repurposed antiviral drugs for COVID-19; interim WHO SOLIDARITY trial results*, medRxiv 2020.10.15.20209817 (2020).

110 See Health Canada, *Search for Clinical Information on Drugs and Medical Devices*, 2019, https://clinical-information.canada.ca/search/ci-rc (accessed Jun. 30, 2020); Health Canada, *The Drug and Health Product Register*, 2019, https://hpr-rps.hres.ca/static/content/about-propos.php (accessed Jun. 30, 2020).

111 See *Food and Drugs Act,* R.S.C. 1985, F-27.

112 See *Food and Drugs Act,* R.S.C. 1985, F-27, s 3.3.

113 See Health Canada, *Public Release of Clinical Information: Guidance Document*, *supra* note 36.

114 See Health Canada, *The Drug and Health Product Register*, 2019, https://hpr-rps.hres.ca/static/content/about-propos.php (accessed Jun. 30, 2020).

115 See *Astrazeneca Canada Inc v Health Canada*, (2005) FC 189 at 76, aff’d 2006 FCA 241.

116 It is worth noting that the USFDA publishes information about the progress of post-market studies. However, these data are often out of date and provide limited insight into the design of the post-market studies. See Joshua D. Wallach et al., *Postmarket studies required by the US Food and Drug Administration for new drugs and biologics approved between 2009 and 2012: cross sectional analysis*, 361 BMJ (2018), https://www.bmj.com/content/361/bmj.k2031 (last visited Oct. 17, 2020).

117 See Jason W Nickerson & Matthew Herder, *Covid-19 Vaccines as Global Public Goods*, in Vulnerable: The Law, Policy, and Ethics of COVID-19 591 (Coleen M Flood et al., eds, 2020). W. Nicholson Price, Arti K. Rai & Timo Minssen, *Knowledge transfer for large-scale vaccine manufacturing*, 369 Science 912–914 (2020).

118 It is important to note that the compulsory licensing provision lapsed on Sept. 30, 2020. Presumably, Parliament could renew that provision swiftly if and when it is warranted. See *Patent Act*, R.S.C. 1985, c. P-4, s 19.4.

119 See *Merck Frosst Canada Ltd v Canada (Health),* (2012) SCC 3; and, W. Nicholson Price, Arti K. Rai & Timo Minssen, *Knowledge transfer for large-scale vaccine manufacturing*, 369 Science 912–914 (2020).

120 TRIPS: Agreement on Trade-Related Aspects of Intellectual Property Rights, Art. 39(3) Apr. 15, 1994, Marrakesh Agreement Establishing the World Trade Organization, Annex 1C, 1869 U.N.T.S. 299, 33 I.L.M. 1197 (1994).

121 The text of the Guidance Document reads: Furthermore, ‘any drug that is issued an authorization under the Interim Order cannot be used as a Canadian Reference Product for the purposes of a submission made under the Regulations in which a drug compares itself to a drug authorized under the Interim Order.’ Additionally, the Interim Order does not allow an application from another drug based on a direct or indirect comparison to an innovative drug, unless the manufacturer for the innovative drug is unable to meet the Canadian market demand in sufficient quantities. Prior to allowing an application under such circumstances, Health Canada will invite the manufacturer of the innovative drug to make representations. (emphasis added) See September Interim Order: Guidance Document, *supra* note 29.

122 See Health Canada, *Public Release of Clinical Information: Guidance Document*, *supra* note 36.

123 It appears that there is growing hesitancy in Canada and the US about COVID-19 vaccines. See Jung, Hauser et al., *supra* note 90. For reason related to vaccine hesitancy in general, see Eve Dubé et al., *Understanding Vaccine Hesitancy in Canada: Results of a Consultation Study by the Canadian Immunization Research Network*, 11 Plos One e0156118 (2016).

124 See Brit Trogen B. et al., *Adverse Consequences of Rushing a SARS-CoV-2 Vaccine: Implications for Public Trust*, 323(24) J.A.M.A. 2460 (2020), DOI: 10.1001/jama.2020.8917.

