# Supplementary material for: Transparency too little, too late? Why and how Health Canada should make clinical data and regulatory decision-making open to scrutiny in the face of COVID-19
Source: J Law Biosci. 2020 Nov 19;7(1):lsaa083. doi: 10.1093/jlb/lsaa083 (PMC7717257; doi:10.1093/jlb/lsaa083)
Supplement: Transparency_too_late_Supplementary_File_FINAL_lsaa083 [file transparency_too_late_supplementary_file_final_lsaa083.docx]

**Supplementary File**

**COVID-19 Interventions Currently in Development in Canada**

*Note:* All information extracted from clinicaltrials.gov, current as of July 15, 2020

| **Identifier** | **Intervention(s)** | **Primary Outcome Measures** | **Responsible Party** | **Start Date** | **Completion Date** | **# of Participants** |
| --- | --- | --- | --- | --- | --- | --- |
| ***Drugs*** | | | | | | |
| NCT04330690  Canadian Arm of Solidarity Trial | Lopinavir/ritonavir  Remdesivir  Hydroxychloroquine | All-cause mortality, measured at hospital discharge | Sunnybrook Health Sciences Centre | June 2020 | March, 2022 | 2900 |
| NCT04321993 | Lopinavir/ritonavir  Hydroxychloroquine  Baricitinib (janus kinase inhibitor) | Clinical status at Day 15 (on a 7-point ordinal scale) | Lisa Barrett, Nova Scotia Health Authority | April, 2020 | March 2022 | 1000 |
| NCT04321174 | Lopinavir/ritonavir | Evidence of infection, adverse events; symptomatic COVID-19, seropositivity; days of hospitalization; respiratory failure requiring ventilatory support; Mortality | Darrell Tan  St. Michael's Hospital, Toronto | April 2020 | March 2022 | 1220 |
| NCT04329611 | Hydroxychloroquine | Composite of hospitalization, invasive mechanical ventilation or death within 30 days; mortality; Symptom duration | Michael Hill  Alberta Health Services  University of Alberta | April 2020 | September 2020 (Trial currently suspended) | 1660 |
| NCT04431453 | Remdesivir | Incidence of adverse events; Incidence of Laboratory Abnormalities | Gilead Sciences | June 2020 | December 2020 | 52 |
| NCT04374942 | Hydroxychloroquine | Incidence of microbiologically confirmed COVID-19 cases | Megan Landes  University Health Network, Toronto | April 30, 2020 | January 2022 | 988 |
| NCT04371523 | Hydroxychloroquine | Number of Healthcare Workers that test positive for SARS-CoV-2 | Waleed Al-Hazzani  St. Joseph’s Healthcare Hamilton | May 2020 | August 30, 2020 | 1100 |
| NCT04347798 | Hydroxychloroquine | Impact of anti-malarials on the development and severity of COVID-19 | University of Alberta | September 2020 | April 2021 | 500 |
| NCT04421664 | Hydroxychloroquine | 3-point Ordinal Scale of COVID-19 Disease Severity at 14 days | Todd Lee  McGill University Health Centre/Research Institute | March 2020 | August 2020 | 1500 |
| NCT04308668 | Hydroxychloroquine | Number of participants at 14 days post enrollment with active COVID-19 disease | University of Minnesota  (Canadian Collaborator: McGill University Health Centre/Research Institute) | March 2020 | May 2020 | 1309 |
| NCT04383002 | Nitric Oxide | COVID-19 PCR status at completion of treatment (day 7) | University Health Network, Toronto | July 2020 | December 2020 | 20 |
| NCT04415060 | Isoflurane Inhalant Product  Sevoflurane Inhalant Product | Hospital Mortality (2 years), Ventilator-Free Days | Sunnybrook Health Sciences Centre | June 2020 | June 2022 | 752 |
| NCT04467086 | Propranolol Hydrochloride | Primary sedative dose change (Change from baseline in total daily dose on Day 3) | James Downar  Ottawa Hospital Research Institute | September 2020 | March 2021 | 108 |
| NCT04402957 | LSALT Peptide | Development of Acute Respiratory Distress Syndrome (28 days) | Arch Biopartners Inc. | July 2020 | June 2021 | 60 |
| NCT04382924 | NP-120 (Ifenprodil) | Patient clinical status (7-point ordinal scale) at day 15 | Algernon Pharmaceiticals | July 2020 | February 2022 | 682 |
| NCT04397692 | Nitric Oxide, delivered by proprietary medical device | Time to deterioration (14 days) | Beyond Air Ltd. | June 2020 | September 2020 | 20 |
| NCT04356677 | Virazole | Change in clinical status severity (7-point ordinal scale) at day 7 | Bausch Health Americas, Inc. | September 2020 | April 2021 | 50 |
| NCT04362137 | Ruxolitinib | Proportion of patients who die, develop respiratory failure, or require intensive care (29 days) | Novartis Pharmaceuticals | May 2020 | October 2020 | 402 |
| NCT04322682 | Colchicine | Number of participants who die or require hospitalization due to COVID-19 infection (30-days post randomization) | Montreal Heart Institute | March 2020 | September 2020 | 6000 |
| NCT04448119 | Favipiravir | Control of outbreak, defined as no new cases of COVID-19 for 24 consecutive days (up to 40 days) | Appili Therapeutics Inc. | June 2020 | March 2021 | 760 |
| NCT04320615 | Actemra/RoActemra (tocilizumab) | Clinical status assessed using 7-point ordinal scale (28 days) | Hoffman-La Roche Ltd. | April 2020 | September 2020 | 450 |
| NCT04337918 | NORS (Nitric Oxide Releasing Solution) | COVID-19 infection among healthcare professionals at risk (14 days)  Efficacy of reducing infection among those infected (21 days) | Sanotize Research and Development Corp. | May 2020 | September 2020 | 200 |
| NCT04443868 | Nitric Oxide-Releasing Drug | Number of subjects requiring hospitalization or ER visits for COVID-19-like symptoms (28 days) | Sanotize Research and Development Corp. | September 2020 | July 2021 | 300 |
| NCT04350593 | Dapagliflozin | Time to first occurrence of either death or new/worsened organ dysfunction (randomization through 30 days) | Saint Luke’s Health System | April 2020 | December 2020 | 900 |
| NCT04354259 | Peginterferon Lambda- 1A | Proportion of participants with negative COVID-19 tests (7 days); Rate of serious adverse events (up to 30 days) | University Health Network, Toronto | May 2020 | November 2020 | 140 |
| NCT04417257 | LAU-7b | Health status of the patient on the 7-point ordinal scale on day 14 | Laurent Pharmaceuticals Inc. | June 2020 | January 2021 | 300 |
| NCT04375735 | Bovine Lipid Extract Surfactant | Adverse events - decrease in oxygenation or hemodynamics (3 days post-randomization) | Lawson Health Research Institute | July 2020 | July 2021 | 20 |
| NCT04401150 | Vitamin C | Death or persistent organ dysfunction, assessed at 28 days | Universite de Sherbrooke | June 2020 | January 2022 | 800 |
| NCT04435795 | Ciclesonide | Improvement in dyspnea at day 7 | Nicole Ezer  McGill University Health Centre/Research Institute | June 2020 | March 2021 | 454 |
| NCT04405102 | Ozanimod | Mean oxygen flow required to maintain the 92% oxygen saturation (7 days); Patient progression on adapted 6-point ordinal scale (14 days) | Francois Lellouche  Laval University | July 2020 | July 2022 | 48 |
| NCT04372589 | Heparin | Intubation and mortality (30 days) | University of Manitoba | May 2020 | January 2021 | 3000 |
| NCT04324463 | Chlorquine hydroxychloroquine azithromycin interferon beta-1b (Betaseron) | Composite of hospitalization or death; invasive mechanical ventilation or death (45 days post randomization) | Population Health Research Institute | April 2020 | June 2021 | 4000 |
| NCT04412018 | Icosapent ethyl (Vascepa) | Change in hs-CRP levels, randomized from day 1 to 14 | Canadian Medical and Surgical Knowledge Translation Research Group | June 2020 | December 2020 | 100 |
| NCT02735707 | lopinavir/ritonavir (Kaletra)  interferon beta-1a (Rebif)  hydroxychloroquine  Heparin Sodium (unfractionated heparin), Lovenox (enoxaparin sodium), Fragmin (dalteparin sodium), Innohep (tinzaparin sodium),  frozen plasma from recovered COVID-19 patients (Convalescent Plasma)  Anakinra  Vitamin C | All-cause mortality (90 days); Days alive and outside of ICU (21 days) | MJM Bonten, UMC Utrecht | April 2016 | December 2023 | 7100 |
| NCT04331665 | Ruxolitinib | Proportion of patients with COVID-19 pneumonia who become critically ill (6 months); Number of adverse events (9 months) | University Health Network, Toronto | April 2020 | January 2021 | 64 |
| ***Biologicals*** | | | | | | |
| NCT04377568 | Convalescent Plasma | Clinical Recovery Time, defined by normal respiratory and heart rate in last 24 hours | Julia Upton  The Hospital for Sick Children, Toronto | July 2020 | May 2022 | 100 |
| NCT04348656 | Convalescent Plasma | Intubation or death in hospital (within 30 days) | McMaster University (Hamilton Health Sciences Corporation) | May 2020 | December 2020 | 1200 |
| NCT04442048 | IMM-101 | Rate of flu-like illness, as defined by WHO (1-year time frame) | Canadian Cancer Trials Group | July 2020 | March 2021 | 1500 |
| NCT04400032 | Mesenchymal Stromal Cells | Number of patients with treatment-related adverse events (up until 1-year post-infusion) | Ottawa Hospital Research Institute | May 2020 | June 2021 | 9 |
| NCT04439045 | VPM1002  (Other Name: Recombinant Mycobacterium bovis rBCGΔureC::hly) | Incidence of COVID-19 infection (7 months) | University Health Network, Toronto | June 2020 | June 2021 | 3626 |
| NCT04401475 | EB05 | Improvement of two points on the 7-point ordinal scale (28 days) | Edesa Biotech Inc. | August 2020 | April 2021 | 865 |
| NCT04376684 | Otilimab | Proportion of participants alive and free of respiratory failure at Day 28 | GlaxoSmithKline | May 2020 | December 2020 | 800 |
| NCT04362085 | Therapeutic Anticoagulation | Composite outcome of ICU admission, non-invasive positive pressure ventilation, mechanical ventilation, or all-cause death (28 days) | St. Michael’s Hospital, Toronto | May 2020 | December 2020 | 462 |
| ***Vaccines*** | | | | | | |
| NCT04398147 | Recombinant Novel Coronavirus Vaccine (Adenovirus Type 5 Vector) | Incidence of adverse events; Incidence of serious adverse events | CanSino Biologics Inc.  The Beijing Institute of Biotechnology and the Canadian Centre for Vaccinology | August 2020 | December 30, 2021 | 696 |
| NCT04334980 | bacTRL-Spike Vaccine for Prevention of COVID-19 | Frequency of Adverse Events; Immune response against SARS-CoV-2 Spike protein; Incidence of COVID-19 infection | Symvivo Corporation | July 2020 | December 2021 | 112 |
| NCT04380532 | Tableted COVID-19 Therapeutic Vaccine | Effect on CBC as per CTCAE v4.0; Effect on biochemistry parameters as per CTCAE v4.0; Lack of adverse events as per CTCAE v4.0 | Immunitor LLC | May 2020 | June 15, 2021 | 20 |
| NCT04450004 | Intramuscular Vaccine | Immediate adverse events (30 minutes); solicited adverse events (7 days); Unsolicited adverse events (21 days); safety labs (3 days); neutralizing antibody response (21 days) | Medicago | July 2020 | April 2021 | 180 |
